# Efficacy of an immunotoxin to folate receptor beta in the intra-articular treatment of antigen-induced arthritis

**DOI:** 10.1186/ar3831

**Published:** 2012-05-02

**Authors:** Taku Nagai, Akira Kyo, Kazuhisa Hasui, Sonshin Takao, Takami Matsuyama

**Affiliations:** 1 Department of Immunology, Graduate school of Medical and Dental Sciences, Kagoshima University, Kagoshima 890-8544, Japan; 2 Cancer and Regenerative Medicine, Frontier Science Research Center, Kagoshima University Graduate School of Medical and Dental Sciences, Kagoshima 890-8544, Japan

## Abstract

**Introduction:**

We previously demonstrated that synovial sublining macrophages express folate receptor beta (FRβ). The aim of this study was to evaluate the efficacy of intra-articular administration of a recombinant immunotoxin to FRβ for treating rat antigen-induced arthritis.

**Methods:**

A monoclonal antibody (mAb) to rat FRβ was produced by immunizing mice with B300-19 cells (murine pre-B cells) transfected with the rat FRβ gene. Recombinant immunotoxin was prepared by conjugating the Fv portion of the anti-rat FRβ mAb heavy chain with a truncated *Pseudomonas exotoxin A *and the Fv portion of the anti-rat FRβ mAb light chain. Antigen-induced arthritis was induced through intra-articular injection of methylated bovine serum albumin (mBSA) after two subcutaneous injections of mBSA and complete Freund's adjuvant. Immunotoxin was intra-articularly injected into the arthritis joint every other day for seven days after arthritis onset. Joint swelling was measured and histological scores of inflammation, synovial thickness, cartilage, and bone destruction were determined. Immunohistochemistry was performed to detect osteoclast and osteoclast precursor FRβ-expressing macrophages and cathepsin K-positive cells on day 21.

**Results:**

Intra-articular administration of the immunotoxin attenuated joint swelling (61% suppression; *P *< 0.01 compared to the control on day 21) and improved histological findings, particularly cartilage and bone destruction (scores of rats treated with control versus the immunotoxin: 2.2 versus 0.5; *P *< 0.01), by reducing the number of FRβ-expressing macrophages and cathepsin K-positive cells.

**Conclusions:**

Intra-articular administration of an immunotoxin to FRβ is effective for improving rat antigen-induced arthritis.

## Introduction

Rheumatoid arthritis (RA) is a chronic, systemic inflammatory disease characterized by synovial hyperplasia and excessive mononuclear cell infiltration in the synovium leading to cartilage and bone tissue degradation. Macrophages are the primary cell type involved in RA synovitis pathogenesis by producing TNF-α, a primary activator of macrophages; differentiation of macrophages to osteoclasts results in bone destruction [1,2]. Clinical disease activity in RA is strongly correlated with the number of macrophages in synovial tissues [3,4], and anti-TNF biological agents are thought to target synovial sublining macrophages [5]. Thus, selective counteraction of synovial macrophage activation remains an attractive approach for diminishing local and systemic inflammation as well as for preventing irreversible joint damage.

We previously reported that synovial sublining macrophages express folate receptor beta (FRβ) as a receptor for oxidized folate [6,7]. Interestingly, these FRβ-expressing macrophages predominantly expressed M1 macrophage markers [8]. Because FRβ expression is limited in normal tissues, we hypothesized that removing FRβ-expressing macrophages may be useful for treating RA and minimize adverse side effects. We previously demonstrated that the activity of RA synovium engrafted in severe combined immunodeficiency (SCID) mice was reduced following administration of an immunotoxin to FRβ around the synovium [9]. Furthermore, an immunotoxin to FRβ prevented osteoclast formation in RA synovial macrophage cultures. In agreement with our previous study, several studies showed that RA synovial FRβ-expressing macrophages may be potential targets for treating RA, utilizing the folate receptor (FR) as the drug delivery system [10,11].

Some RA patients develop monoarthritis and oligoarthritis during very early stages. Additionally, despite a good response of other joints to systemic administration of anti-TNF biologics in combination with disease-modifying anti-rheumatic drugs, many patients continue to experience persistent symptoms in a single or a few joint(s) [12]. Intra-articular drug administration, radiation, or surgical synovectomy can be very useful for treating disease flare-ups, synovitis, and pain when a small number of joints are affected or in patients with joints that do not respond to systemic medications [13-16]. Indeed, intra-articularly administered corticosteroids, which are commonly used for treating RA with monoarthritis and oligoarthritis, show superior effectiveness and tolerance compared with systemic corticosteroid use. However, the effect of corticosteroids is not permanent. Furthermore, some arthritic joints are refractory to intra-articular corticosteroid injection, and additional drugs are not routinely available. A number of studies evaluating intra-articular anti-TNF injections have shown variable efficacies of this treatment [17]. Therefore, intra-articular administration of drugs with different mechanisms of action may be necessary for use as local RA therapy.

In this study, we evaluated the efficacy of intra-articular administration of a recombinant immunotoxin to FRβ for treating rat antigen-induced arthritis (AIA).

## Materials and methods

### Production of anti-rat FRβ monoclonal antibody (mAb)

Rat FRβ cDNA was prepared from a product derived from Lewis rat liver using (RT-PCR). Primer sequences used were 5'-tctagaaagacatggcctggaaacag-3' (forward) and 5'-cccaacatggatcaggaact-3' (reverse). B300-19 (murine pre-B) cells transfected with the rat FRβ gene were prepared as previously described [9,18]. Balb/c mice were immunized using rat FRβ gene-transfected B300-19 cells. Lymphocytes from iliac lymph nodes and spleen lymphocytes were fused with NS-1 myeloma cells. Hybridomas were screened for their reactivity with rat FRβ gene-transfected B300-19 cells. One anti-rat FRβ mAb (4A67, immunoglobulin M (IgM)) was selected for further evaluation. All animal studies were performed in accordance with the Ethical Guidelines for Animal Experiments of Kagoshima University (approval number: MD09074 & MD10099).

### Production of a recombinant immunotoxin to rat FRβ

Mouse Ig 4A67 cDNA was obtained using RT-PCR with primers from the Ig-prime kit (Novagen, Madison, WI, USA). Sequences were deposited in GenBank (accession number JN588994 for IgV_H_, JN588993 for IgV_L_). Recombinant variable region fragment antibody (Fv) constructs were produced as previously described [9,18]. Briefly, cysteine residues were introduced into the variable regions of the Ig heavy chain (IgV_H_) gene (Gly40Cys) and the light chain (IgV_L_) gene (Gly105Cys) using PCR and the Quick Change Site-Directed Mutagenesis kit (Stratagene, La Jolla, CA, USA). To generate plasmid constructs of these mutated genes, PCR was performed using the following primers: for the IgV_H _gene, 5'-CATATGcagatccagttggtgcagtctgga-3' (upstream) and 5'-tccggAAGCTTttgaggagacggtgactgaggttcc-3' (downstream); for the Ig V_L _gene, 5'-taagaaggagatataCATATGcaaattgttctcacccagtct-3' (upstream) and 5'-gctttgttagcagccGAATTCctattttatttccaactttgtcccacagccgaacgt-3' (downstream). These primers introduced restriction enzyme sites (underlined) for cloning of the IgV_H _gene into the *Nde*I-*Hin*dIII site and the IgV_L _gene into the *Nde*I-*Eco*RI site in the pUli7 expression vector. The IgV_H _gene was ligated with a truncated *Pseudomonas exotoxin A *(PE38) gene and IgV_L _gene and the resultant plasmid was expressed in *Escherichia coli *BL21 cells. Recombinant immunotoxin (dsFv anti-FRβ-PE38), which consists of the Fv portion of the anti-rat FRβ mAb heavy chain with PE38 (V_H_-PE38) and the Fv portion of the anti-rat FRβ mAb light chain, was prepared as previously described [9,18]. Briefly, inclusion bodies from bacteria transfected with expression plasmids encoding the Ig V_H_-PE38 and IgV_L _genes were solubilized separately and then combined. Properly folded dsFv anti-FRβ-PE38 was purified using HiTrap Q (Amersham Pharmacia, Piscataway, NJ, USA) and Poros HQ (Applied Biosystems, Tokyo, Japan) anion-exchange chromatography and using TSK 3000 SW (Tosoh, Tokyo, Japan) size-exclusion chromatography. To prepare monomeric IgV_H_-PE38 (cysteine residue modification) protein, refolded IgV_H_-PE38 protein was reduced using 1 mM dithiothreitol, alkylated using 10 mM iodoacetamide, and purified as described above. Endotoxin concentrations of recombinant proteins were measured using the *Limulus *amebocyte lysate assay (Associates of Cape Cod, Falmouth, MA), USA. Concentrations were less than five endotoxin units per mg.

### Flow cytometric analysis

Female Lewis rat (six to nine weeks old) macrophages were harvested using peritoneal lavage. Peritoneal exudate macrophages were collected on day 4 after intraperitoneal injection with 10 mL of 3% thioglycollate (TGC). Macrophages were collected using lavage and cultured in 10-cm tissue culture plates in Iscove's Modified Dulbecco's Media (IMDM) containing 10% FCS. After removing non-adherent cells, macrophages were resuspended in IMDM media. Flow cytometric analysis was performed as previously described [7]. Briefly, the cells were reacted with anti-FRβ or isotype-matched control mAbs, followed by allophycocyanin-conjugated goat (Fab')_2 _anti-mouse IgM (R&D Systems, Minneapolis, MN, USA) and phycoerythrin-conjugated murine anti-rat CD11 b/c (OX42; BioLegend, San Diego, CA, USA). Stained cells were analyzed using a FACSAria flow cytometer (Becton Dickinson, Franklin Lakes, NJ, USA).

### Detection of dead cells

TGC-injected macrophages, B300-19 cells, and rat FRβ gene-transfected B300-19 cells were incubated in the presence of V_H_-PE38 or dsFv anti- FRβ-PE38. Dead cells (sub G_0_/G_1 _cells) were detected using propidium iodide staining and flow cytometric analysis as previously described [9,18]. Briefly, cells were stained with propidium iodide (50 μg/mL) in 0.1% sodium citrate plus 0.1% Triton X-100 for 20 minutes at 4°C. Dead cells were detected using flow cytometry.

### Measurement of nitric oxide (NO) produced by peritoneal macrophages

TGC-elicited peritoneal macrophages (2 × 10^5^) were placed in 24-well plates in the presence of either V_H_-PE38 or dsFv anti-FRβ-PE38 and further incubated for 24 hours at 37°C in 5% CO_2_. The medium was exchanged and cells were stimulated using 100 U/mL of IFN-γ (Peprotec, London, UK) and 100 ng/mL of Lipopolysaccharide (LPS ) (Sigma-Aldrich, Tokyo, Japan) for an additional 24 hours. NO in the culture supernatants was measured in duplicate using the Griess reaction.

### Induction of AIA

All experimental rats were immunized using two subcutaneous injections (day -21 and day -14) of 0.5 mg methylated BSA (mBSA) dissolved in 0.05 mL of saline and emulsified in an equal volume of complete Freund's adjuvant [19,20]. Knee monoarticular arthritis was induced on day 14 after the second immunization (day 0 of AIA). The left knee joint was challenged using intra-articular injection of 0.05 mL of saline with 0.5 mg of mBSA, while the right knee joint received 0.05 mL of saline. Arthritis development was monitored at regular intervals by measuring knee diameters using a caliper.

### Treatment with an immunotoxin to rat FRβ

On day 1 of AIA, rats were randomly assigned to one of four groups and intra-articularly injected with 0.05 mL of either V_H_-PE38 (50 µg) or dsFv anti-FRβ-PE38 (2 µg, 10 µg, or 50 µg) every other day until day 7. In all cases, the right knee joint remained untreated. To assess the effect of these treatments, joint swelling was expressed as the difference in diameters between the right and left knees.

### Conventional histology and immunohistochemistry

Rat normal tissues were prepared as cryostat sections and fixed in cold acetone. Untreated knee joints of rats were removed on days 2, 5, 7, 14, and 21 after AIA induction to examine FRβ-expressing macrophages. Treated knee joints of rats were removed on day 21 after AIA induction. Knee joints were fixed in cold acetone, decalcified in 0.5 M ethylenediaminetetraacetic acid (EDTA), immersed in 20% sucrose-PBS, and embedded in 50% optimal cutting temperature (OCT) compound. Cryostat sections (7 µm) were prepared using adhesive film (FINETEC and Leica Microsystems, Tokyo, Japan) [21]. To evaluate histological arthritis scores, the sections were stained using H & E. All slides were evaluated by an independent observer who was blinded to the design and details of the study. Sections were graded as previously described by Williams *et al*. [20] based on the following three parameters: degree of cartilage destruction and bone erosions (0 to 3), severity of synovial infiltration and inflammatory exudates (0 to 3), and degree of synovial membrane thickening (0 to 2).

Immunohistochemistry was performed as previously described [9,18]. Briefly, 7-µm tissue sections were treated using 0.6% H_2_O_2 _for 10 minutes to quench endogenous peroxidase. Sections were blocked using blocking solution (Protein Block Serum-Free; DAKO, Tokyo, Japan). To detect CD68 and FRβ in rat normal tissues in Table 1, sections were reacted with a mixture of mouse IgG_1 _and IgM mAbs as an isotype-matched control; mouse anti-rat CD68 mAb (IgG_1_) (AbD Serotec, Oxford, UK); mouse anti-rat FRβ mAb (IgM) and followed by anti-mouse MAX-PO secondary Ab (Nichirei Co. LTD., Tokyo, Japan). To detect E-cadherin, sections were reacted with biotinylated goat polyclonal Ab as an isotype-matched control; biotinylated goat anti-mouse (cross-reactive to rat) E-cadherin Ab (R&D Systems) and followed by streptovidin-peroxidase (Invitrogen, Tokyo, Japan). To detect FRβ in arthritis joints in time course, sections were reacted with mouse IgM mAb as an isotype-matched control; mouse anti-rat FRβ mAb (IgM) and followed by anti-mouse MAX-PO secondary Ab. To detect CD68, FRβ and cathepsin K in arthritic joints, sections were reacted with a mixture of mouse IgG_1 _and IgM mAbs, and rabbit IgG as an isotype-macthed control; mouse anti-rat CD68 mAb; mouse anti-rat FRβ mAb; rabbit anti-cathepsin K Ab (Biovision, San Franscisco, CA, USA) and followed by anti-mouse MAX-PO secondary Ab.

**Table 1:** Distribution of FRβ in normal rat tissues analyzed using flow cytometry or immunohistochemistry

Flow Cytometric Analysis	FRβ	CD11 b/c
*TGC-elicited Peritoneal mϕ *	87 ± 3.1%	95 ± 2.5%
Immunohistochemical Analysis	FRβ	CD68
		
*Spleen*		
White pulp	-	++
Red pulp mϕ	+	+++
		
*Lymph node *		
Cortex, tingible body mϕ	-	++
Paracortical mϕ	-	+
Medullary mϕ	-	+
Subcapsular sinus mϕ	-	+
		
*Brain *mϕ	-	+
		
*Liver *mϕ (Kupffer cells)	+	++
		
*Lung *		
Perivascular/peribronchial mϕ	-	+
Alveolar mϕ	-	+
		
*Kidney*		
Mesangial mϕ	-	+
		
*Heart*		
Myocardial mϕ	-	+
Endocardial mϕ	-	+
		
*Colon*		
Mucosa		
Epithelial mϕ	-	+
Lamina propria mϕ	+	+
Muscularis mucosa mϕ	*-*	+
Submucosal mϕ	*-*	+
Muscularis externa mϕ	*-*	+

Frequency of cell staining (-, <100/10 fields), (+, 100-200/10 fields), and (++, 200-300/10 fields), (+++, >300/10 fields). Data was obtained from three normal rat tissues with similar results. FRβ, folate receptor β; TGC, thioglycollate.

These sections were visualized using a DAB staining kit (DAKO). Sections were then counter-stained using haematoxylin. Images were obtained using a digital sight CCD camera (DS-Fi1; Nikon, Tokyo, Japan). Semi-quantitative analyses were carried out using a computer-aided image analyzer (NIS-Elements; Nikon). Brown-stained cells in rat normal tissues and the synovial membrane and subchondral bone of ten randomly selected ×400 fields from each section were counted. A color threshold mask for immunostaining was defined to detect brown color by sampling. This threshold was applied for all samples.

### Statistical analysis

Statistical analyses were performed using the nonparametric Mann-Whitney *U*-test. Data are expressed as mean ± standard error. A value of *P *< 0.05 was considered statistically significant.

## Results

### Specificity and reactivity of anti-rat FRβ mAb

**Figure 1:**
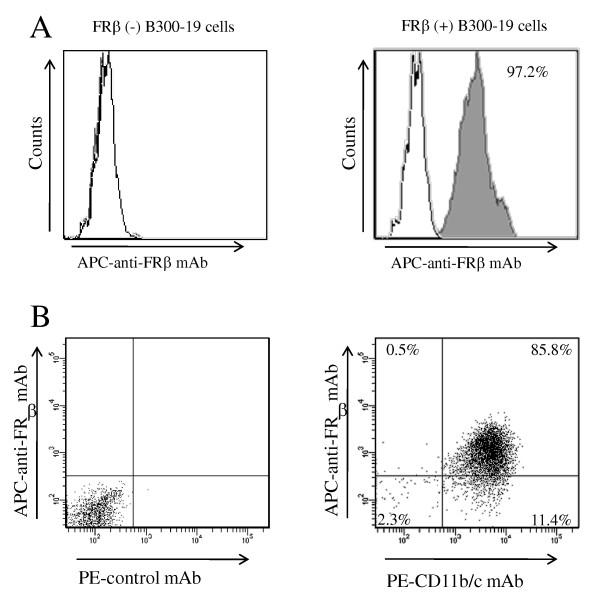
**Reactivity of an anti-rat FRβ mAb with FRβ-expressing cells**. **(**A**) **Non-transfected B300-19 cells (left) and rat FRβ gene-transfected B300-19 cells. (right) were stained using an anti-rat FRβ (4A67) mAb (black pattern) or isotype-matched irrelevant mAb (white pattern). Stained cells were analyzed using flow cytometry. Data are representative of three separate experiments. (**B**) TGC-elicited macrophages were double-stained using an anti-FRβ mAb (4A67) and PE-conjugated CD11b/c mAbs (OX42) and analyzed using flow cytometry. Numbers represent the percentage of cells within designated gates. Data are representative of three separate experiments. FRβ, folate receptor β; mAb, monoclonal antibody; TGC, thioglycollate.

**Figure 2:**
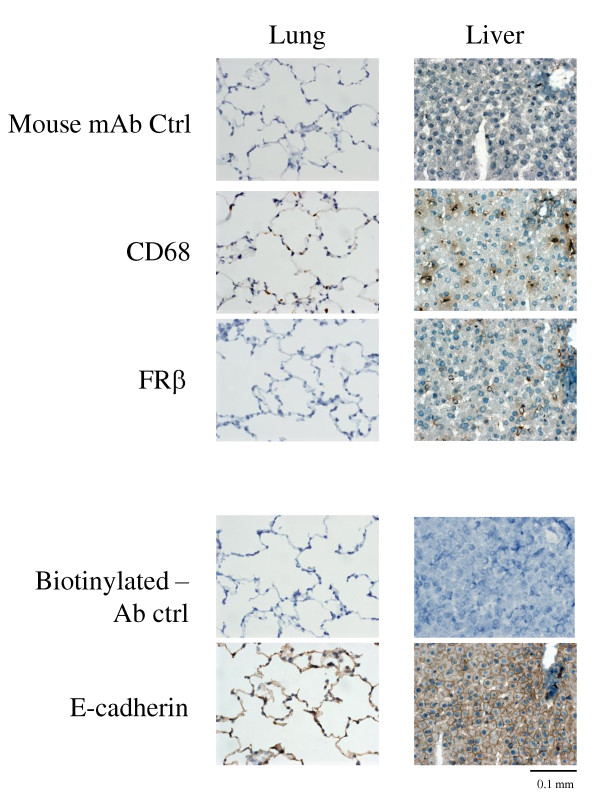
**Reactivity of an anti-rat FRβ mAb with rat lung and liver tissues**. Lung and liver tissues from normal rats were stained using antibodies against CD68, FRβ, or E-cadherin. Photographs are representative of CD68-, FRβ-, and E-cadherin staining in lung or liver tissues of three rats per group. Note that FRβ-positive cells were observed in the liver but not in the lung. CD68- positive cells and E-cadherin-positive cells (indicating the presence of epithelial cells) were observed in both tissues; original magnification was ×400. FRβ, folate receptor β; mAb, monoclonal antibody.

Antibody clone 4A67 reacted with rat FRβ gene-transfected cell lines (97.3 ± 0.3%) but did not react with the original cell lines (1.5 ± 0.2%) (Figure 1A). Most TGC-elicited peritoneal macrophages highly expressed FRβ (87 ± 3.1%) (Figure 1B and Table 1). As shown in Table 1, the distribution of FRβ in rat tissues was similar to that in murine tissues reported previously [18]. Figure 2 shows FRβ-expressing macrophages of the rat lung as an example of negative staining and the liver as an example of positive staining. E-cadherin-positive cells were observed in both tissues, indicating the presence of epithelial cells. Thus, it appears that the anti-FRβ antibody did not react with FRα, which is known to be preferentially expressed on the apical surface of the epithelia in human lungs, kidneys, and intestinal tissues [22].

### **Effects of dsFv anti-rat FRβ immunotoxin on apoptosis and NO production in FRβ-expressing cells**

**Figure 3:**
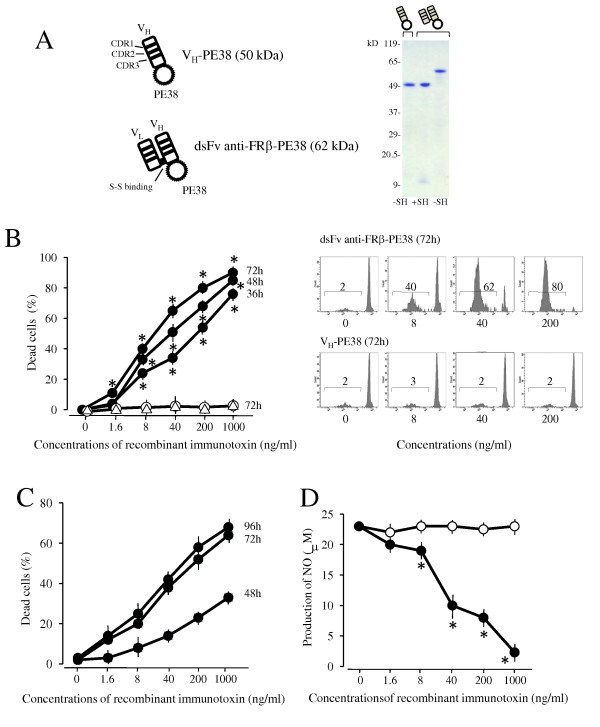
**Effects of anti-FRβ immunotoxin on cell death induction and NO production of FRβ-expressing cells**. **(A)**The schema shows the construction of V_H_-PE38 and dsFv anti-FRβ-PE38. The purity of V_H_-PE38 and dsFv anti-FRβ-PE38 were analyzed using sodium dodecyl sulphate- 6% to 15% gradient gel electrophoresis in non-reducing (-SH) and reducing conditions (+SH). Molecular weight markers are shown at left. (**B**) Rat FRβ-transfected B300-19 cells were cultured at the indicated concentrations of V_H_-PE38 (open triangle) for 72 hours or at the indicated concentrations of dsFv anti-FRβ-PE38 (filled circle) for 36, 48, or 72 hours. Non-transfected B300-19 cells were also cultured at the indicated concentrations of dsFv anti-FRβ-PE38 (open circle) for 36, 48, or 72 hours. The number of dead cells was measured using propidium iodide staining and flow cytometric analysis, and the percentage of dead cells was calculated as the number of dead cells divided by total cell number. Values are the mean ± SEM of induced cell death from three separate experiments. The right panels are representative of propidium iodide staining patterns at the indicated concentrations of V_H_-PE38 or dsFv anti-FRβ-PE38. Values in panels show the percentages of dead cells. (**C**) Thioglycollate-elicited macrophages were cultured at the indicated concentrations of V_H_-PE38 or dsFv anti-FRβ-PE38 for 48, 72, or 96 hours. Induced cell death (%) was determined by subtracting the percentage of dead cells induced by V_H_-PE38 from that induced by dsFv anti-FRβ-PE38 in each sample. Values are the mean ± SEM of induced cell death of three separate experiments. (**D**) Thioglycollate-elicited macrophages were cultured at the indicated concentrations of V_H_-PE38 (open circle) or dsFv anti-FRβ-PE38 (filled circle) for 24 hours and stimulated using IFN-γ and LPS for an additional 48 hours. NO in the supernatants was measured using the Griess reaction. Values are the mean ± SEM of three separate experiments. * *P *< 0.05 compared to the V_H_-PE38 group. FRβ, folate receptor β; IFN-γ, interferon-γ; LPS, Lipopolysaccharide**; **SEM, standard error of the mean.

We produced recombinant dsFv anti-rat FRβ immunotoxin composed of the Fv portion of the light chain and the Fv portion of the heavy chain with PE38 as described in the Methods section. Figure 3A shows the construction of an immunotoxin and the patterns of purified V_H-P_PE38 and the immunotoxin by SDS-PAGE. A 62-kDa band and a faint lower band corresponding to the immunotoxin and a 50-kDa band corresponding to V_H-_PE38 were observed under non-reducing conditions; 50-kDa and 12-kDa bands of the immunotoxin were observed under reducing conditions. To assess the *in vitro *efficacy of this immunotoxin, we evaluated whether the immunotoxin could induce death of rat FRβ gene-transfected cells and TGC-elicited peritoneal macrophages. The immunotoxin induced death of rat FRβ gene-transfected B300-19 cells and TGC-elicited peritoneal macrophages with IC_50 _values of 10 ng/mL and 100 ng/mL, respectively, but did not change that of the original B300-19 cells (Figure 3B and 3C). Additionally, the immunotoxin suppressed NO production by peritoneal macrophages activated using IFN-γ and LPS at lower concentrations (35.4 ± 2.1 ng/mL at 50% suppression) than those inducing cell death (Figure 3D).

### Suppression of joint swelling of arthritis by dsFv anti-FRβ immunotoxin

**Figure 4:**
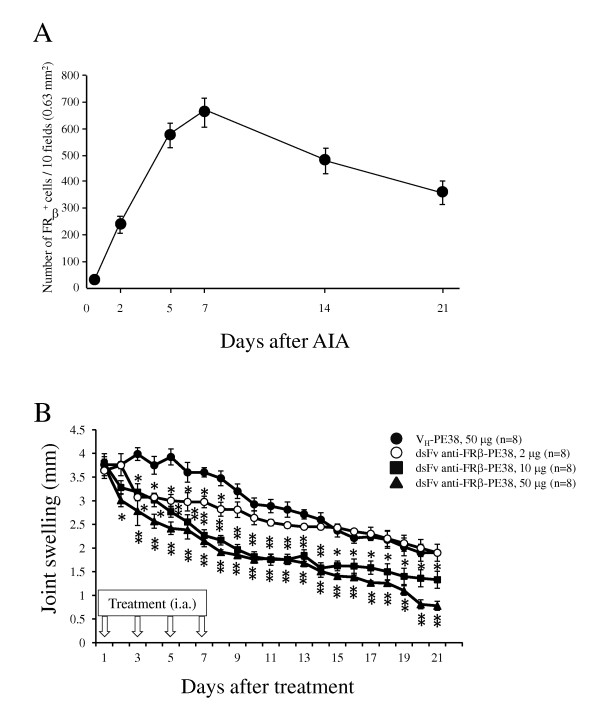
**Effects of anti-FRβ immunotoxin on knee joint swelling in rat AIA**. (**A**) FRβ-expressing macrophages in synovium of the arthritis rat were examined on the indicated days after AIA onset as described in Methods. Values are the mean ± SEM of knee joints from four rats per group. (**B**) AIA was induced using intra-articular injection of methylated BSA as described in Methods. Arthritic rats were injected in the left knee joint with 50 μg of V_H-_PE38 (filled circle) or with 2 µg (open circle), 10 µg (filled square) or 50 μg (filled triangle) of dsFv anti-FRβ-PE38 on days one, three, five and seven. Knee joint swelling was measured for each rat by measuring the difference in diameter between the arthritic left knee and the normal right knee. Values are the mean ± SEM of knee joint swelling from eight rats per group. *P <0.05 and **P <0.01 compared to the V_H-_PE38-treated group. AIA, antigen-induced arthritis; BSA, bovine serum albumin; FRβ, folate receptor β; SEM, standard error of the mean.

A previous study showed infiltration of macrophages in the synovium of AIA [23]. Similarly, we observed peak infiltration of FRβ-expressing macrophages in arthritic synovium from days five through seven (Figure 4A). Arthritic joints were treated intra-articularly using three different doses of immunotoxin. These treatments reduced joint swelling over the course of therapy in a dose-dependent manner compared to that using the control protein (V_H_-PE38) (Figure 4B). Treatment with medium and high doses resulted in a reduction in joint swelling even at 14 days post-injection. The degree of suppression was 26.7 ± 3.2% (*P *< 0.05) at the medium dose and 61.6 ± 3.4% (*P *< 0.01) at the high dose compared to the control on day 21. Body weight was 174 ± 4 g (rats treated with control) and 173 ± 5 g (rats treated with high-dose immunotoxin) on day 0, and 203 ± 8 g (rats treated with control) and 198 ± 11 g (rats treated with high-dose immunotoxin) on day 21. Changes in body weight between the groups were not significant at any time points.

### Improvement in histological scores in arthritic joints following intra-articular administration of dsFv anti-FRβ immunotoxin

**Figure 5:**
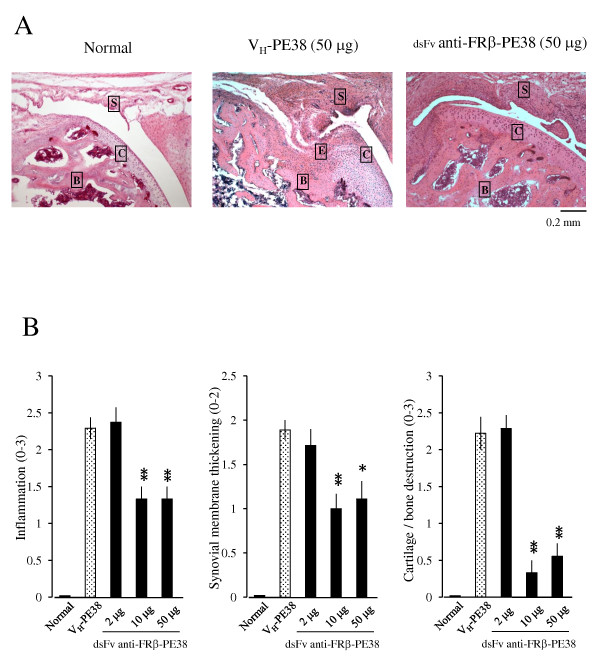
**Effects of an anti-FRβ immunotoxin on the histology of knee joints in rat AIA**. (**A**)Photographs are representative of knee joints from eight rats per group. Knee joints from normal rats and AIA rats treated with 50 µg of V_H_-PE38 or dsFv anti-FRβ-PE38 were stained using H & E. Note that a knee joint from an AIA rat treated using dsFv anti-FRβ-PE38 showed less cartilage/bone destruction compared to a rat treated using V_H_-PE38. Letters S, C, B, and E represent synovium, cartilage, bone, and erosion. Original magnification was ×100. (**B**)Histological scores were recorded for inflammation, synovial membrane thickening, and cartilage/bone destruction. Data are presented as the mean ± SEM of each histological score from eight rats per group. *P < 0.05 and **P < 0.01 compared to the group treated using V_H_-PE38. AIA, antigen-induced arthritis; FRβ, folate receptor β; H & E, haematoxylin and eosin; SEM, standard error of the mean.

**Figure 6:**
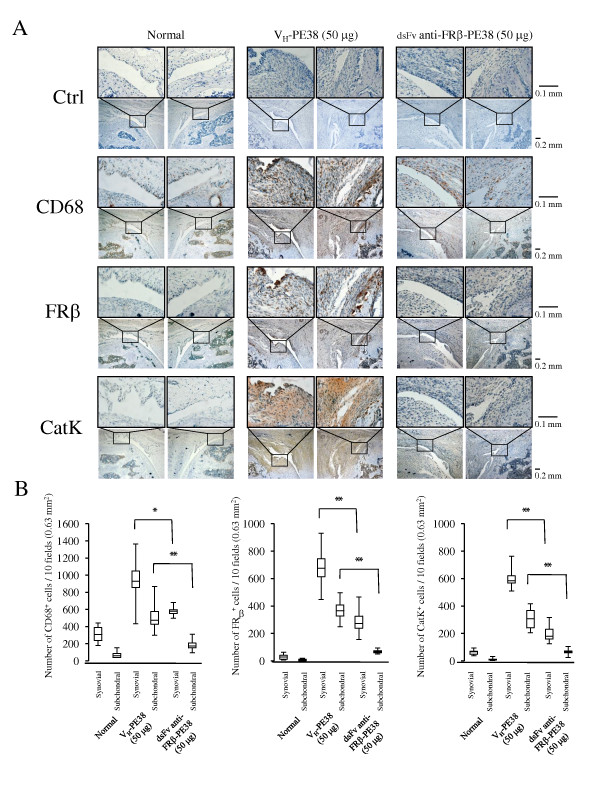
**CD68-, FRβ-, and cathepsin K-positive cells in the knee joints of AIA rats treated using an anti-FRβ immunotoxin**. (**A**) Knee joints from normal rats and AIA rats treated with 50 µg of V_H_-PE38 or dsFv anti-FRβ-PE38 were stained with isotype-matched controls (a mixture of mouse IgG_1 _and IgM mAbs, and rabbit IgG), antibodies against CD68, FRβ, or cathepsin K, and positive cells were counted. Photographs are representative of negative control and CD68-, FRβ-, and cathepsin K-positive cells in the knee joints of eight rats per group. Note that the synovium from AIA rats treated using dsFv anti-FRβ-PE38 showed fewer CD68-, FRβ-, and cathepsin K-positive cells compared to those treated using V_H_-PE38. Original magnifications were ×100 and ×400. (**B**)Numbers of CD68-, FRβ-, and cathepsin K-positive cells were counted in the knee joints of each group. Data from eight rats per group are presented as box plots, where the boxes represent the 25th to 75th percentiles, the lines within the boxes represent the median, and the lines outside the boxes represent the 10th and 90th percentiles. *P < 0.05 and **P < 0.01 compared to the group treated using V_H_-PE38. AIA, antigen-induced arthritis; FRβ, folate receptor β; Ig, immunoglobulin.

Histopathological evaluation was performed at the end of the study (day 21). As shown in Figure 5A and 5B, arthritic joints treated using medium and high doses showed lower grades of inflammation, synovial membrane thickness, and cartilage and bone destruction compared to those with control protein treatment or treatment with a low dose of immunotoxin. The effects of immunotoxin were prominent with regard to histological scores, indicating cartilage and bone destruction (scores of rats treated with control versus immunotoxin; 2.2 ± 0.2 versus 0.5 ± 0.1, *P *< 0.01). Thus, we determined the numbers of synovial macrophages, FRβ-expressing macrophages, and cathepsin K-positive cells, which are osteoclast precursors or osteoclasts in synovial and subchondrial regions. Immunohistological analysis revealed that high-dose treatment reduced these numbers, particularly with regard to the numbers of FRβ-expressing macrophages and cathepsin K-positive cells, compared to that with the control protein (Figure 6A and 6B).

## Discussion

We produced an anti-rat FR-β mAb and its dsFv recombinant immunotoxin consisting of the Fv portion of an anti-FRβ mAb and PE38. Using this antibody, it was previously demonstrated that expression and distribution of FRβ-expressing macrophages in rat tissues were similar to those in murine tissues in which FRβ-expressing macrophages are rarely present [18]. After the onset of mBSA-induced arthritis, intra-articular injections of immunotoxin reduced joint swelling during the acute phase of arthritis. Antigen-induced arthritis shows several clinical and histopathological similarities to human RA. Its maximal clinical activity, two to three days post-induction, chronic arthritis develops, characterized by synovial hyperplasia, inflammatory inflammation, and cartilage and bone destruction [24]. Destruction of cartilage matrix results predominantly from the action of connective tissue proteinase released by RA synovial tissues, chondrocytes, and pannus tissues. Several lines of evidence in RA and animal models of arthritis support a role for osteoclasts in the pannus tissue in the pathogenesis of bone erosions. RA synovial tissues, including macrophages, fibroblasts, and T cells, produce a variety of cytokines and growth factors that may increase osteoclast formation, activity, and/or survival. Specifically, the interaction of the receptor activator of nuclear factor-kappaB ligand (RANKL) produced by fibroblasts and T cells on RANK on osteoclast precursors is critical for inducing the differentiation of cells of the monocyte/macrophage lineage into osteoclasts [25]. We previously observed that most osteoclasts originate from FRβ-expressing macrophages in RA synovial culture and that administration of dsFv anti-FRβ-PE38 reduced the number of activated fibroblasts and macrophages in RA synovial tissue engrafted into SCID mice [8]. Thus, removal of macrophages using the immunotoxin may reduce the number of osteoclast precursors and osteoclasts, protecting against cartilage and bone destruction in arthritis.

Findings in rat arthritis models indicate that osteoclasts are formed soon after the onset of clinical arthritis and are continuously replenished by infiltrating macrophages during disease progression [26]. Thus, early and effective blockade of osteoclastogenesis may be accomplished by targeting infiltrated macrophages. In this study, we showed that intra-articular injection of medium- and high-dose immunotoxins reduced the number of FRβ-expressing macrophages, even at 14 days post-injection. Several studies have demonstrated that systemic administration of FR-mediated drugs is effective for reducing the activity of experimental arthritis [9,11,27-29]; however, it remains unknown whether intra-articular administration of these drugs is effective for treating arthritic joints. This is the first study demonstrating that intra-articular injections targeting FRβ-expressing macrophages are efficacious as local therapy.

Intra-articular injections of corticosteroids or hyaluronate are generally available for treating mono and oligoarthritis [14,15,30]. Although these treatments appear to be safe and beneficial, the reduction in pain and inflammation is only temporary. Consistent with these clinical outcomes, it has been reported that macrophage infiltration and proinflammatory endothelial cytokine expression remained unchanged in arthroscopic biopsies of RA synovial tissues after intra-articular administration of corticosteroids [31]. Additionally, intra-articular administration of high-molecular weight hyaluronate promoted joint swelling, inflammation, and cartilage damage during the late chronic phase of mBSA-induced arthritis [32]. Thus, intra-articular injections of immunotoxin appear to be effective for improving long-term effects compared to the use of corticosteroids and hyaluronate. Several studies have demonstrated that intra-articular injections of anti-TNF biologics are effective for treating RA or experimental arthritic joints, although cumulative data of intra-articular administration of available biologics to RA joints show inconsistent levels of effectiveness [17,33]. Additionally, local therapy using anti-TNF biologics has not been evaluated in arthritis refractory to systemic anti-TNF therapy because many patients in successful cases were naïve to systemic anti-TNF biologics. Experimental studies have demonstrated that anti-TNF biologics are capable of binding membranous TNF on macrophages and mediate antibody-dependent cell-mediated cytotoxicity and complement-dependent cytotoxicity [34,35]. An anti-FRβ immunotoxin induces apoptosis of FRβ-expressing macrophages by PE38 to inhibit protein synthesis by elongation factor 2 [36]. However, we did not find a significant difference of ratios of apoptotic cells detected by tunnel staining in synovial macrophages between treated and non-treated synovium on day21 (data not shown). We speculate that apoptotic cells induced by the immunotoxin may be removed during the 14 days that had passed since the last injection Thus, intra-articular injection of immunotoxin may be useful for treating arthritis refractory to systemic anti-TNF therapy. Cartilage penetration by proteins is dependent on a protein's molecular weights and charges; full-length IgG cannot penetrate cartilage [37]. The dsFv anti-FRβ immunotoxin is of lower molecular weight (62 kDa) than full-length IgG, allowing higher penetration into synovial tissues than full-length IgG. Interestingly, single-chain Fv anti-TNFα mAb inhibited acute inflammation of the knee joint induced by intra-articular administration of recombinant human TNF-α, although this model was not directly representative of RA. Conversely, a lower molecular weight may have a lower half-life and fewer prolonged effects [38]. In fact, we did not detect the immunotoxin in sera and synovium from treated mice on the last day (data not shown). These results are reasonable because it was reported that the half-life of DsFv-PE38 immunotoxin in sera is less than 30 minutes [39].

To increase retention time in joints, intra-articular injections of immunotoxins using nanoparticles and/or liposomes may be beneficial for clinical application [40]. Clinical studies have shown that immunotoxins based on PE38 show lower nonspecific toxicity but retain antigen-dependent toxicity towards target cells [41]. Local injection can generally be effective at lower doses than systemic injection. Thus, intra-articular administration of immunotoxin may overcome several challenges, including non-specific cytotoxicity and immunogenicity resulting from systemic usage of the immunotoxin.

## Conclusions

Intra-articular administration of an immunotoxin to FRβ was effective for improving rat antigen-induced arthritis. This treatment may provide a new strategy for targeting limited arthritis with rheumatoid arthritis in which FRβ-expressing macrophages are abundant.

## Abbreviations

AIA: antigen-induced arthritis; BSA: bovine serum albumin; FCS: fetal calf serum; FR: folate receptor; Fv: fragment of Ig consisting of heavy: light chain variable domains; H & E: haematoxylin and eosin; IFN: interferon; Ig: immunoglobulin; IMDM: Iscove's Modified Dulbecco's medium; mAb: monoclonal antibody; PBS: phosphate-buffered saline; PE38: truncated *Pseudomonas exotoxin-*A - should be removed; RA: rheumatoid arthritis; RANKL: receptor activator of nuclear factor-kappaB ligand; RT-PCR: reverse transcriptase-polymerase chain reaction; SCID: severe combined immunodeficiency; TGC: thioglycollate; TNF: tumor necrosis factor; V_H_: heavy chain variable domain; V_L_: light chain variable domain;

## Competing interests

The authors declare that they have no competing interests.

## Authors' contributions

TN and AK carried out animal experiments, and statistical analysis. TN, KH and TM carried out the immunohistology and statistical analysis. TN, AK, KH, ST, and TM participated in the design and analysis of the study and helped to draft the manuscript. All authors read and approved the final manuscript.
